# Mechanism and Kinetics of Hydration of CuSO_4_·H_2_O in the Presence of an Intermediate Step

**DOI:** 10.1021/acs.cgd.4c00589

**Published:** 2024-12-09

**Authors:** Martina Cotti, Amelie Stahlbuhk, Hartmut R. Fischer, Michael Steiger, Olaf C. G. Adan, Henk P. Huinink

**Affiliations:** † Eindhoven Institute of Renewable Energy Systems, Eindhoven University of Technology, PO Box 513, Eindhoven 5600 MB, The Netherlands; ‡ Transport in Permeable Media group, Department of Applied Physics, Eindhoven University of Technology, PO Box 513, Eindhoven 5600 MB, The Netherlands; § Department of Chemistry, University of Hamburg, Martin-Luther-King-Platz 6, Hamburg 20146, Germany; ∥ TNO Materials Solution, High Tech Campus 25, Eindhoven 5656 AE, The Netherlands

## Abstract

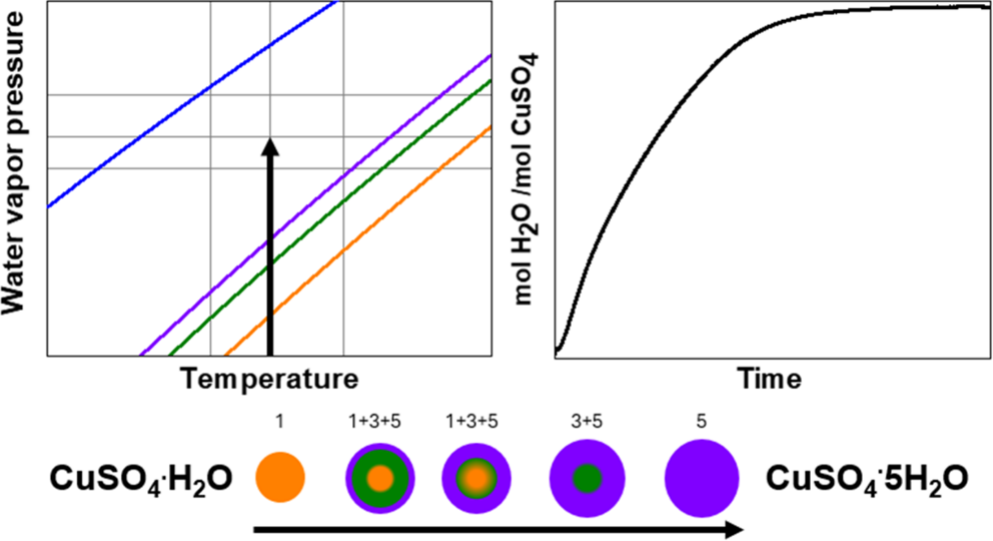

The hydration of salt hydrates is often described as a solution mediated nucleation and growth mechanism, occurring between a reagent and a product in thermodynamic equilibrium with each other. If a system possesses more than one hydrate phase, the kinetic pathway may involve additional mechanisms due to the formation of intermediate hydrate species. We elected CuSO_4_ as our model system and analyzed the pathway leading from CuSO_4_·H_2_O (C1H) to CuSO_4_·5H_2_O (C5H), while CuSO_4_·3H_2_O (C3H) being a possible intermediate. We found that C1H hydration is mediated by the formation of C3H and that C5H does not nucleate directly from C1H, at the studied conditions. The hydration pathway therefore is characterized by the same mechanism occurring twice, nucleation and growth of C3H and nucleation and growth of C5H. Analysis of the hydration kinetics of C1H revealed that C5H nucleates rapidly from C3H, as if the metastability of C3H was reduced when starting from C1H. Therefore, we concluded that the hydration kinetics of C1H are probably controlled by the growth process of C5H. Despite being controlled by a single reaction process, we show that a single front 1D diffusion model is insufficient to describe the reaction kinetics at the tablet level. Understanding of these complex transformations is necessary to evaluate the suitability of these reactions for application, in particular with respect to the achieved power output.

## Abstract 2

The hydration pathway of CuSO_4_·H_2_O to CuSO_4_·5H_2_O is mediated by the formation the intermediate CuSO_4_·3H_2_O. Direct hydration from CuSO_4_·H_2_O to CuSO_4_·5H_2_O was not observed. The hydration kinetics of C1H are influenced by the metastability of CuSO_4_·3H_2_O and by the growth of product phases on top of the reagent phases as in a shrinking core configuration.

## Introduction

Salt hydrates can be used as thermochemical materials for storing and releasing heat through reversible binding of water vapor.^1−3^ As long as the water and the salt are kept separate, loss-free storage can be achieved. However, it is known that the speed of hydration—the speed of discharge—of these materials may be low. Thermal gravimetric hydration measurements of milligram amounts of salt powder show that half-conversion times vary between several minutes and hours, depending on the salt used and the conditions imposed. Furthermore, the power output usually rises and drops dramatically within few minutes from the start of the reaction, which is undesirable from the application point of view.^4−10^

The hydration mechanism of salts needs to be understood to achieve faster hydration and more stable power outputs. A hydration reaction is a phase transition in between any couple of hydrated phases of a specific salt. In most cases two phases are involved in the transition.^11,12^ According to the general description, hydration is constituted by two processes: nucleation and growth.^11,13−16^ It was elaborated that nucleation of the product phase limits the hydration kinetics at low supersaturations and in the so-called metastable zone.^11,14^ Instead, growth of the product phase limits the hydration kinetics at high supersaturations.^10,13,17,18^

It is unclear how nucleation and growth control the hydration kinetics in the case of systems with more than one hydrated phase. In these systems, the applied reaction conditions may force the reagent phase to cross the stability region of another phase, and consequently the kinetic pathway may require the formation of an intermediate. Hydrations across multiple phases are already exploited in several application studies.^17,−22^ In fact, higher energy densities can be reached by storing more water molecules, and hydrating across multiple phases can be beneficial.^1^ Therefore, it is clearly relevant to expand our fundamental understanding of these more complex transformations.

These transformations are usually characterized by unprecedented kinetic behavior due to the possible formation of intermediates.^23^ For example, Blijlevens et al.^24^ observed the formation of only one intermediate (only the dihydrate, not the monohydrate) during thermal gravimetric measurements of SrCl_2_ hydration, from the anhydrous to the hexahydrate phase. Similarly, Ferchaud et al. did not observe the formation of CuSO_4_·3H_2_O when hydrating CuSO_4_·H_2_O to CuSO_4_·5H_2_O.^25^ According to the work of Molenda et al.,^26^ the intermediate phase of CaCl_2_ is either monohydrate or one-third hydrate, depending on the direction of the scan. The hydration of MgSO_4_·H_2_O to MgSO_4_·7H_2_O observed through X-ray diffraction displayed a prolonged metastability of the intermediate hexahydrate phase.^27^ Finally, the hydration of Y_2_(SO_4_)_3_ (0–8) was deemed nonstoichiometric by the authors.^28^ Due to the presence of intermediates, hydration may be controlled by multiple processes, for example by nucleation and growth at the same time. Consequently, the reaction rate may need to be described by more complex expressions.^15^

We decided to study the transitions between the hydrates of CuSO_4_ for the wide availability of starting knowledge. The structure,^29−34^ the thermodynamics^35^ and the decomposition mechanisms^25,36−39^ are well characterized. From the field of catalysis, there is a wealth of research investigating how water molecules organize around the copper cation in the liquid and solid state.^40−42^ Its application as thermochemical material presents opposing views, as the energy density of the system is relatively large (1.93 GJ m^–3^), but the temperature lift is relatively low (5–10 °C), depending on the temperature of the water source.^43,44^ Experimental studies on prototypes featuring CuSO_4_ have been recently published.^45,46^

**Figure 1:**
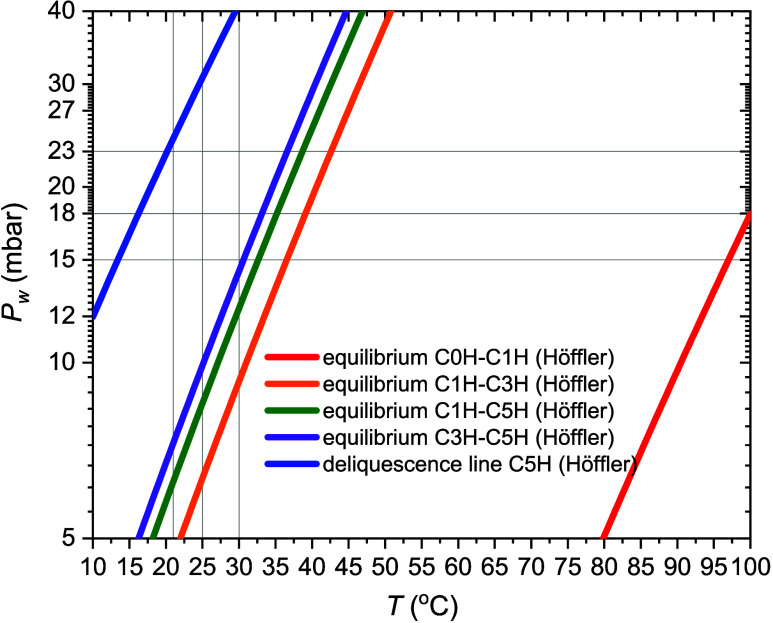
Phase diagram of the system CuSO_4_-water. All these lines have been obtained from eq 3 using the thermodynamic quantities calculated by Höffler.^35^

In this paper we identify the sequence of mechanisms defining the hydration pathway of CuSO_4_·H_2_O (C1H) to CuSO_4_·5H_2_O (C5H) and elaborate on the steps and main processes dictating the reaction kinetics. According to the phase diagram (Figure 1), there is a thermodynamic equilibrium between C1H and C3H, as well as a metastable equilibrium between C1H and C5H.^35^ Therefore, there are in total two pathways that potentially lead to the formation of C5H from C1H based on the following three reactions
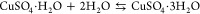
1.1
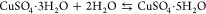
1.2
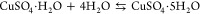
2the first, through the formation of the intermediate C3H (pathway 1), the second leading directly to C5H (pathway 2). Pathway 3 instead is used to address the direct formation of C5H from pure C3H.

To study the kinetics of these reactions, the hydration onsets—water vapor pressure and temperature—are determined by thermal gravimetric analysis (TGA) and electrochemical impedance spectroscopy (EIS). The position of these onsets in the phase diagram identifies a metastable zone (MSZ) line, which generates a framework of conditions used to discriminate in between nucleation and growth processes. Based on the MSZ, hydration kinetic measurements are performed by TGA at various conditions, and the shapes, and the time scales of these curves are used to obtain insight on the occurrence of nucleation and growth process. Details of the phase transformation are obtained by in situ analysis of the reaction by Raman spectroscopy (RS) and X-ray diffraction (XRD). Our results combined allow us to determine the sequence of processes occurring during the hydration of C1H and to hypothesize which of these processes is limiting the overall hydration rate.

## Theory

The phase diagram of CuSO_4_ in Figure 1 reports the equilibrium conditions–equilibrium water vapor pressure (*p*_eq_) and temperature (*T*)—at which two phases coexist with water vapor. The *p*_eq_ can be calculated from Clausius–Clapeyron equation^47^ for a dehydration reaction
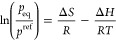
3where *p*^ref^ is equal to 1 bar, Δ*S* (J mol^–1^ K^–1^) is the standard entropy of dehydration per mole of water, Δ*H* (kJ mol^–1^) is the standard enthalpy of dehydration per mole of water and *R* (J mol^–1^ K^–1^) is the ideal gas constant.

At a water vapor pressure *p*_w_ > *p*_eq_, the higher hydrate phase is stable, below *p*_eq_ the lower hydrate phase is stable. CuSO_4_ has three hydrated phases: monohydrate (C1H), trihydrate (C3H) and pentahydrate, (C5H). For example, at 30 °C and based on the equilibrium lines, C1H is the stable phase when *p*_w_ < 9 mbar, C3H when 9 < *p*_w_ < 14 mbar and C5H when *p*_w_ > 14 mbar but lower than the deliquescence water vapor pressure, (*p*_del_), (blue line). Due to the C1H–C5H metastable transition, C1H and C5H could also coexist in the stability region of C3H (green line).

The phase diagram also expresses that the system can potentially transform from one to the other phase when *p*_w_ and *T* are in the right region. For example, at 30 °C and 11 mbar, C1H can hydrate to C3H. By increasing the water vapor pressure to 18 mbar, both C1H and C3H can hydrate to C5H. Due to the metastable equilibrium between C1H and C5H, C1H could directly hydrate to C5H when the water vapor pressure is at 12 mbar. However, at the same conditions, C5H may dehydrate to C3H.

As a result of the kinetics of hydration, the thermodynamic potential may not coincide with what is observed. In fact, a specific transformation may not occur instantaneously, in which case the initial phase is said to be metastable.^11,27^ From now on we will use the term metastable or metastability to refer to the kinetics of the reaction, to avoid confusion with the concept of metastable equilibrium.

Typically a minimum driving force (*p*_w_/*p*_eq_ ≫ 1), or supersaturation, is necessary to achieve instantaneous nucleation.^11^ If this driving force is not high enough, the mass does not increase for a period called induction time. Recently it was proven that the induction time is linked to the low ionic mobility of the system.^14^ After nucleation, the product phase can grow. In several instances it was found that the growth rate was dependent on *p*_w_/*p*_eq_. Nucleation and growth are distinct processes, and together they determine a mechanism.

## Materials and Methods

### Materials Preparation

CuSO_4_·5H_2_O (C5H) powder was purchased from Sigma-Aldrich. The powder was ball milled for 30 min in an agate jar and sieved to obtain a 50–150 μm size distribution. This powder was then dehydrated in the oven for several days at 105 and 40 °C, to obtain respectively CuSO_4_·H_2_O (C1H) and CuSO_4_·3H_2_O (C3H). The phase composition was verified by X-ray diffraction. The morphology of the obtained powders was also characterized by scanning electron microscopy (Figure A1). Cylindrical tablets of C1H were produced by using a column press (PO-Weber PW-40 2). The same mass of powder (500 mg) was pressed by 0.85 kbar of pressure to produce tablets of 12.3 ± 0.1 mm in diameter, 2.2 ± 0.1 mm in thickness and 50 ± 1% porosity.

### Thermal Gravimetric Analysis

Thermal gravimetric analysis (TGA) on salt powders was performed on a Mettler Toledo thermal gravimetric analysis (TGA)/DSC3+ instrument connected to a commercial humidifier (Cellcraft). Typical samples weighted 5 mg and were placed in a aluminum 40 μL crucible. Once in the furnace they were flushed by a humidified N_2_ flow at 300 mL min^–1^. The humidifier moisturizes a fraction of the set flow according to the water vapor pressure (*p*_w_) of choice. The wet and dry flow ratios are calibrated by detecting the deliquescence points of LiCl·H_2_O, MgCl_2_·6H_2_O, and K_2_CO_3_·1.5H_2_O at 25 °C. The sample temperature is calibrated by determining the melting points of In, Pb and Zn standards through the heat flow signal under flow of N_2_. In the end of the calibration, the accuracy on the *p*_w_ is 0.2 mbar.

Metastable zone measurements were conducted at constant water vapor pressure and varying furnace temperature at 1 K min^–1^. The temperature was scanned from 105 to 20 °C in the case of CuSO_4_·H_2_O and CuSO_4_·5H_2_O and from 40 to 20 °C in the case of CuSO_4_·3H_2_O. Hydration kinetics measurements were performed in isothermal and isobaric conditions. The mass of the sample was first equilibrated for 2 h, at 105 °C and 5 mbar (CuSO_4_·H_2_O) or at 40 °C and 8 mbar (CuSO_4_·3H_2_O). The temperature was then lowered to the desired measurement temperature (either 21 or 25 °C) and the weight was equilibrated for another hour. At this point, the water vapor pressure was adjusted to the final value. Given the size, a homemade aluminum sample holder was manufactured to host tablets in the TGA. This sample holder was a cup, 16 mm in diameter with 1 mm thick sides. The hydration kinetic measurements of the tablets followed the same technique as for the powder.

### Electrochemical Impedance Spectroscopy

Electrochemical impedance spectroscopy (EIS) was used to probe the ionic mobility in CuSO_4_ hydrates at constant temperature and in the presence of pure water vapor. This application of EIS is described for the first time by Houben et al.^14^ and we refer to this paper for a detailed description of the system. In brief, the set up consists of a vacuum oven, connected to a water vapor generator and equipped with a VersaSTAT 4 Potentiostat Galvanostat from AMETEK. The measurements were done at 25 °C. Water vapor was generated by a water vessel kept at a set temperature by an ethyl glycol bath in a LAUDA chiller. Before water vapor could access the chamber, the oven was evacuated up to a minimum pressure of 0.25 mbar. CuSO_4_ powders (C1H, C3H, and C5H) were pressed into large tablets (*d* ∼ 30 mm, *t* ∼ 3–4 mm and *m* ∼ 6 g) and inserted in the oven in between two electrodes connected to the potentiostat. The temperature of the sample was checked by a thermocouple. During the measurement, the water vapor pressure was increased from 5 to 23–25 mbar in steps of 1 mbar, each 20 min long. Measurements are performed in potentiostatic mode, by applying a constant voltage amplitude across the sample and measuring the resulting current in the frequency range 10^–1^–10^6^ Hz. From the measured impedance, *Z* (Ω), the conductivity, δ (S/m) was calculated, according to
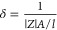
where *A* (m^2^) is the surface of the tablet in contact with the electrodes and *l* (m) is the thickness.

### In Situ Raman Spectroscopy (RS)

The hydration of C1H was observed in situ under a Raman microscope and the phase identification was performed by comparison with literature data.^31^ The set up consists in a Bruker Senterra Raman microscope, an MHG Modular Humidity Generator and a Linkam THMS600 sample chamber. The Raman spectra were obtained by shining on the samples a green laser beam (532 nm) with 20 mW power. The spectra were collected from 50 to 1500 cm^–1^, with a resolution of 3–5 cm^–1^. The integration time was 5 s. Every spectrum was obtained by the coaddition of 5 spectra. The humid flow was generated at 21 °C by mixing a wet and dry N_2_ flow in proportion to obtain humidities from 40 to 85% RH at the sample temperature. The total flow was 500 mL/min. The temperature of the chamber was also controlled and set to 21 °C. Measurements were conducted on a powder with size distribution *d* < 50 μm. The first spectra were acquired before the tubing carrying the N_2_ flow was attached.

### X-ray Diffraction

The phase composition of all powders was checked by X-ray diffraction (XRD) and analyzed with the help of Rigaku PDXL2 software and literature references.^32−34^ The instrument operated was a Rigaku Miniflex 600 X-ray diffractometer (Cu Kα radiation; Be monochromator, λ = 1.5419 Å, 40 kV, 15 mA) equipped with a D/tex Ultra2 1D detector. The milled powders were positioned in the instrument on top of a Nickel sample holder and the diffractograms were collected with a resolution of 0.05° and a speed of 5°/min.

The Rigaku Miniflex diffractometer was equipped with an in situ measurement set up constituted by an Anton-Paar BTS500 heating stage and a home-built humidifier. The sample chamber was flushed by 800 mL/min of humidified N_2_ gas, calibrated based on the hydration transitions of LiCl at 40 °C, 50 and 60 °C.

Hydration kinetics measurements were conducted in isobaric and isothermal conditions. At the start of the measurement the sample chamber was flushed by humid N_2_ either at 5 mbar (CuSO_4_·H_2_O) or 8 mbar (CuSO_4_·3H_2_O), while the sample stage temperature was kept 105 or 40 °C, respectively. The temperature was then lowered to either 25 or 30 °C and after 1 h of equilibration the water vapor pressure was switched to 18 mbar. A diffractogram was measured either every 30 min or every hour.

## Results

### Metastable Zones

**Figure 2:**
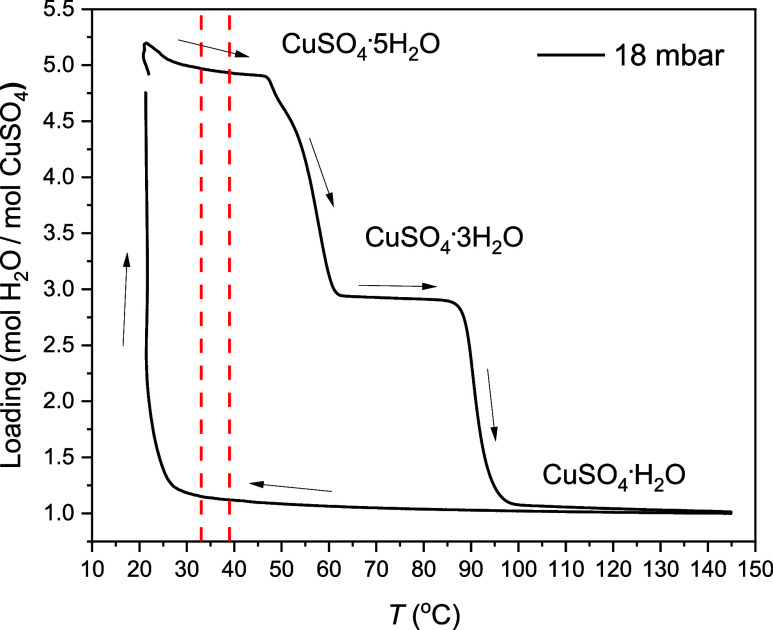
Loading as a function of temperature at 18 mbar during the measurement for the determination of the dehydration and hydration metastable onset temperatures by TGA. The arrows show the direction of the experiment: starting from a powder sample of CuSO_4_·5H_2_O—loading of 5—the loading drops in 2 steps during heating, and it increases in 1 step during cooling. The equilibrium temperatures for the two transitions are indicated by two red vertical lines, at 33 and 39 °C. The temperature was scanned at 1 K min^–1^ from 21 to 145 °C.

The onset temperatures of dehydration and hydration, which define the metastable zones of the reactions, can be measured by detection of a sudden mass increase. These experiments are performed by TGA, by imposing isobaric conditions and scanning the temperature at a fixed rate. The onset temperatures are determined from the intersection of the two tangents to the curve when the mass increases. Figure 2 reports the loading as a function of temperature for one measurement performed at 18 mbar by varying the temperature at a rate of 1 K min^–1^ from 21 to 145 °C. Loading stands for the moles of water molecules per mole of CuSO_4_, therefore in this measurement it varies from 5 (starting point, C5H) to 1 (end point, C1H), and vice versa. These experiments have been performed at other water vapor pressures as well and can be visualized in the Supporting Information.

At all water vapor pressures (Figure A2a) the loading dropped in 2 steps as the temperature was raised, reaching first a loading of 3 and then a loading of 1. During the cooling interval, the loading remained rather constant through most of the temperature range, until it increased back again to the initial value in one step. These results are compatible with previously published isobaric measurements of CuSO_4_ hydration.^25^ However, the loading increases from 3.5 to 4.75 mol mol^–1^ under isothermal conditions, at 21 °C. Therefore, we cannot fully exclude that a second uptake step might appear during cooling if lower temperatures could be accessed. In fact, our experiments are limited to room temperature by the lowest accessible temperature of the device, the temperature of the cooler (18 °C).

**Figure 3:**
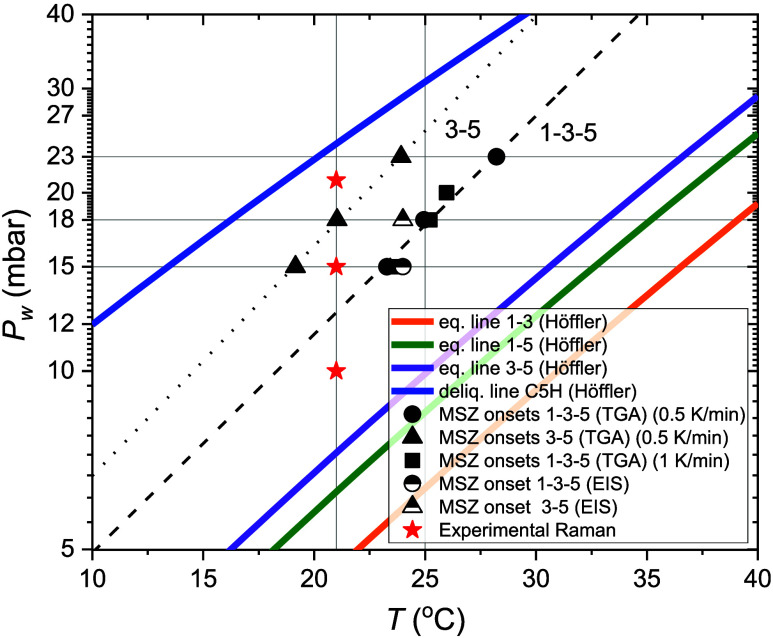
Phase diagram of CuSO_4_-water in terms of water vapor pressure and temperature. The onsets of hydration measured by TGA are shown as black symbols. The dotted and dashed lines represent a linear fit through these points. The onsets measured by EIS are half full symbols. Finally, the red stars indicate where Raman spectroscopy measurements have been conducted.

The measurements were repeated with a focus on the individual transitions. Therefore, CuSO_4_·H_2_O and CuSO_4_·3H_2_O were obtained from CuSO_4_·5H_2_O, as explained in the Materials and methods, and have been used as starting powders (Figure A2). A single hydration onset temperature was found when starting from CuSO_4_·H_2_O (Figure A2b). Since the loading increased continuously and above 3 mol mol^–1^ after the onset, the presence of a second onset temperature should be excluded. A single onset temperature is also found when starting from CuSO_4_·3H_2_O (Figure A2c). The position of the obtained hydration onset temperatures is visualized in Figure 3 as black symbols. These measurements have been conducted also at lower temperature rates (0.5 and 0.3 K min^–1^) confirming the position of the metastable zone boundaries. All onset temperatures for hydration and dehydration are plotted in Figure A2d of the Supporting Information.

**Figure 4:**
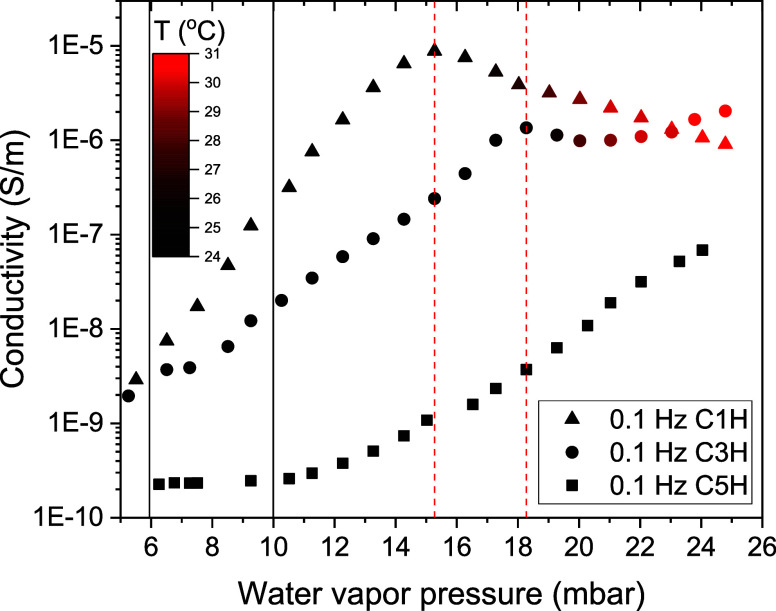
Magnitude of the conductivity at 0.1 Hz plotted as a function of the water vapor pressure for the detection of the metastable zone onset water vapor pressure by EIS. Each symbol represents a hydrate phase. The color of the symbols indicates the temperature of the sample registered simultaneously by a Pt sensor. The onset water vapor pressures are distinguished by a sudden raise in temperature and a simultaneous drop in the admittivity. They are marked by two red vertical lines for C1H at 15 mbar and C3H at 18 mbar and 24 °C. The two black vertical lines mark instead the equilibrium water vapor pressures at 25 °C for the 1–3 (6 mbar) and 3–5 (10 mbar) transitions.

According to Houben et al.,^14^ the presence and width of the hydration MSZ can be determined by analysis of the ionic conductivity in the different hydrates of CuSO_4_. This is done by measurements of the conductivity of a sample in a continuous manner as a function of the frequency (0.1 Hz–1 MHz), while the water vapor pressure is increased stepwise from 5 to 25 mbar at 25 °C. The onset water vapor pressure is recognized by the sudden drop in conductivity and the sudden increase in sample temperature. Figure A3 in the Supporting Information shows the complete conductivity spectra of the three samples (C5H, C3H, and C1H). Since ionic conductivity manifests itself first as a short distance motion, the conductivity at 0.1 Hz is selected from the spectra and plotted as a function of the water vapor pressure in Figure 4.

The admittivity of C5H increased continuously as a function of the water vapor pressure (squares). The admittivity of C1H and C3H, instead, reached a maximum and dropped (triangles and spheres). As indicated by the color of the symbols, the temperature started to increase at the maximum. Based on EIS the onset water vapor pressure is at 15 and 18 mbar at a sample temperature of 24 °C, for C1H and C3H respectively.

The position of these hydration onset temperatures and water vapor pressure with respect to the equilibrium lines is reported in the phase diagram of Figure 3.

Both measurement techniques agreed that all onset points are located to the left of the 3–5 equilibrium line, where CuSO_4_·5H_2_O is stable. The onset temperatures for the hydration of CuSO_4_·H_2_O, found through the two different TGA approaches (full squares and circles) and EIS (half full circle), coincided. The measured onset temperatures defined a single MSZ line. The onsets for the hydration of CuSO_4_·3H_2_O (triangles) occurred at even lower temperatures. The EIS value for this hydration onset (half full triangle) disagreed from the TGA values (full triangles), possibly because C5H impurities in C3H act as nucleation centers and therefore produce a shift of the metastable zone line to lower *p*_w_, during an isothermal measurement.

Therefore, mostly based on TGA data, the two MSZ will be called 1–3–5 when starting from C1H and 3–5 when starting from C3H.

Since the metastability of the monohydrate extended in the region where the pentahydrate is stable, it is not clear whether the monohydrate also transforms directly to the pentahydrate or if the trihydrate is formed as an intermediate. In the next section we are going to determine the order of formation of the different phases by Raman spectroscopy on powder particles. To reduce the number of possible reactions, from now on measurements are conducted above the 3–5 equilibrium line.

### Nucleation on the Surface of Powder Particles

**Figure 5:**
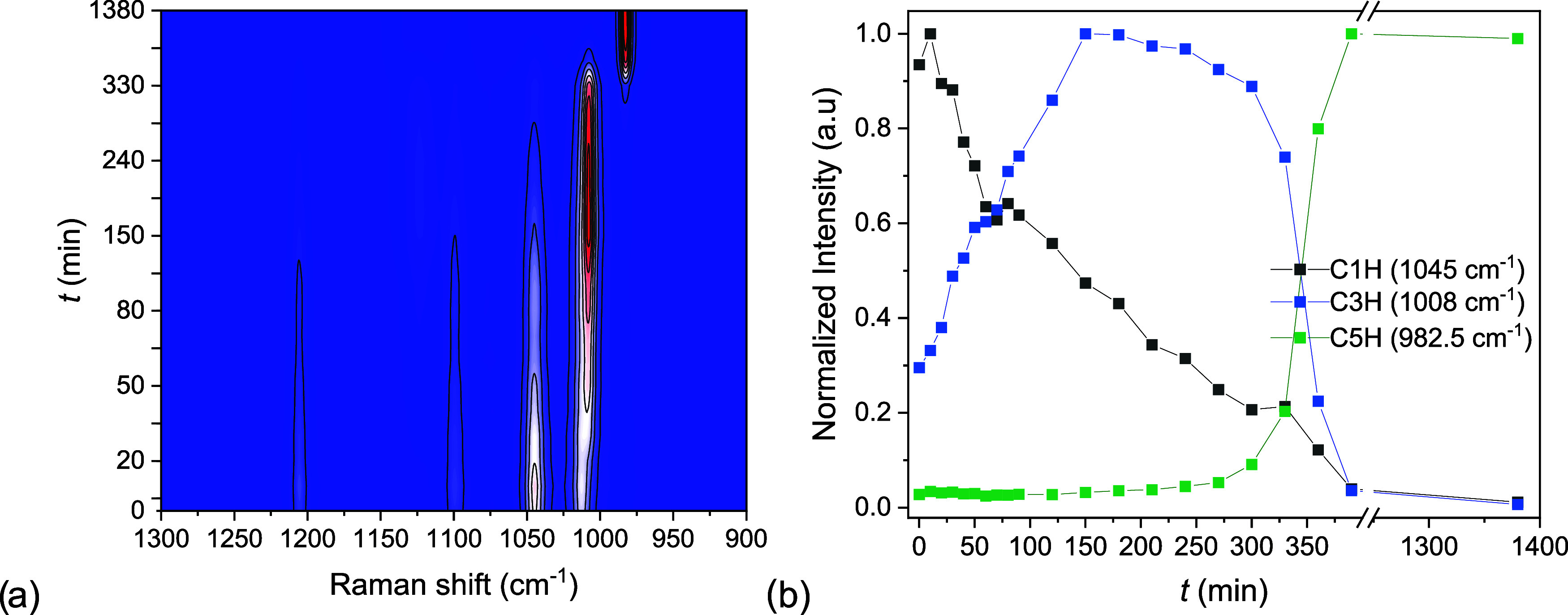
Phase composition study by Raman spectroscopy during hydration of C1H powder samples (*d* < 50 μm) at 21 °C and 85% RH (21 mbar). In (a) the intensity of the Raman spectra changes with the hydration time, from blue to white to red. The peaks used for the analysis are at 1045, 1008, and 983 cm^–1^. In (b) the normalized intensity of these peaks is plotted as a function of time to show qualitatively the appearance and disappearance of each phase. Measurements performed at 65%RH (15 mbar) and 40% RH (10 mbar) are shown in the Supporting Information.

The sequence of formation of C3H and C5H from C1H can be detected by Raman spectroscopy through the analysis of the surface of powder particles. In this experiment, C1H powder particles (*d* < 50 μm) are hydrated in situ at 21 °C and 85%RH (21 mbar), Figure 5a. Measurements performed at 60 and 40% RH (15 and 10 mbar) can be found in the Supporting Information (Figure A4). The phase transformation shows as the evolution in time of the intensity of the symmetrical stretching vibration of the sulfate ion. Since the Raman shift of this vibration mode is different for C1H, C3H and C5H, respectively at 1045, 1008, and 983 cm^–1^^31^ the transition in between phases can be easily detected. In the end, an intensity profile normalized to the maximum intensity is obtained for each vibration, as shown in Figure 5b.

At 85% RH and 21 °C the environmental conditions imposed are outside of any metastable zone (Figure 3). The intensities of the three vibration modes changed in time as in Figure 5b. The intensity at 1045 cm^–1^ (C1H) dropped continuously from the beginning until the end. Simultaneously the intensity at 1008 cm^–1^ (C3H) increased. Note that the intensity at 1008 cm^–1^ did not begin from zero, because of a partial overlap with the 1013 cm^–1^ (C1H) vibration. After the intensity at 1008 cm^–1^ reached its maximum, it remained constant for some time. This delay may be due to the metastability for C3H, which was not expected at these conditions. At *t* = 300 min, the intensity at 983 cm^–1^ (C5H) was also detected for the first time. Within 75 min, this peak reached its maximum intensity while the 1008 cm^–1^ peak dropped to 0.

From this measurement we could draw three main lessons. First, the intermediate phase, C3H, was formed before C5H. Second, the nucleation of C5H was emphasized by the simultaneous drop in C3H intensity, indicating the conversion from C3H to C5H. Third, the direct transformation of C1H into C5H was not detected. Therefore, the 1–3–5 MSZ line presented in the earlier section must indicate the formation of C3H from C1H. The nucleation of C5H, masked in the TGA measurement, clearly shows in this Raman experiment and takes place from C3H.

All these observations were confirmed by the measurement at 21 °C and 15 mbar (Figure A4a). Since this time the applied conditions fall in the boundaries of the 3–5 MSZ, time scales were elongated but the phase sequence was equivalent. C3H appeared first once the intensity of C1H started to decline. Then C3H was metastable for several hours and ultimately the nucleation of C5H occurred simultaneously to the disappearance of C3H after 40 h.

Because the edge of the 1–3–5 MSZ is at lower water vapor pressures than 15 mbar, we did not expect C1H to be metastable. This factor may indicate a slight uncertainty in the control of the environmental parameters. A difference of 2 K is enough to observe large changes as the metastable zone boundaries are very close.

The picture collected so far suggests that the hydration pathway from C1H to C5H is mediated by C3H (pathway 1). To understand the reaction kinetics of this complex hydration, we investigated the hydration kinetics of each of its segments separately. This is achieved in the next section by measurements of the hydration kinetics of C3H and C1H to C5H.

### Hydration Kinetics

Since the hydration of C1H to C5H was characterized by a series of processes, in the present section it is questioned how the sequentiality manifests and affects the hydration kinetics. This is achieved by studying separately the hydration kinetics of the reaction from C3H to C5H (pathway 3) and the reaction from C1H to C5H (pathway 1). The hydration kinetics are investigated by TGA, through isothermal–isobaric measurements at two temperatures and several water vapor pressures. To make sure that C5H is the final product, the measurement conditions are chosen above the 3–5 equilibrium line. In this region it is important to distinguish in between conditions above the 1–3–5 MSZ line and below the 3–5 MSZ line, where only C3H is metastable, and conditions below MSZ 1–3–5, where both C3H and C1H are metastable. In the end conversion times of the two reactions are compared as a function of the driving force to elucidate on the role of the intermediate specie on the hydration time scales of C1H.

### Hydration Kinetics of CuSO_4_·3H_2_O to CuSO_4_·5H_2_O

The hydration kinetics of C3H are presented first (pathway 3), as the reaction from 3 to 5 has appeared to be a component of the more complex reaction from 1 to 5. TGA measurements are shown at two temperatures (21 and 25 °C) and several water vapor pressures. CuSO_4_·3H_2_O was equilibrated first at 40 °C and 8 mbar for 2 h, before the temperature was switched to the measurement temperature. At the equilibration conditions C3H dehydration is metastable, Figure A2. After one more hour of equilibration, the water vapor pressure was adjusted to the final value and this moment was marked as zero. The mass increase was expressed as loading; hence the curves vary from 3 to 5 as a function of time.

**Figure 6:**
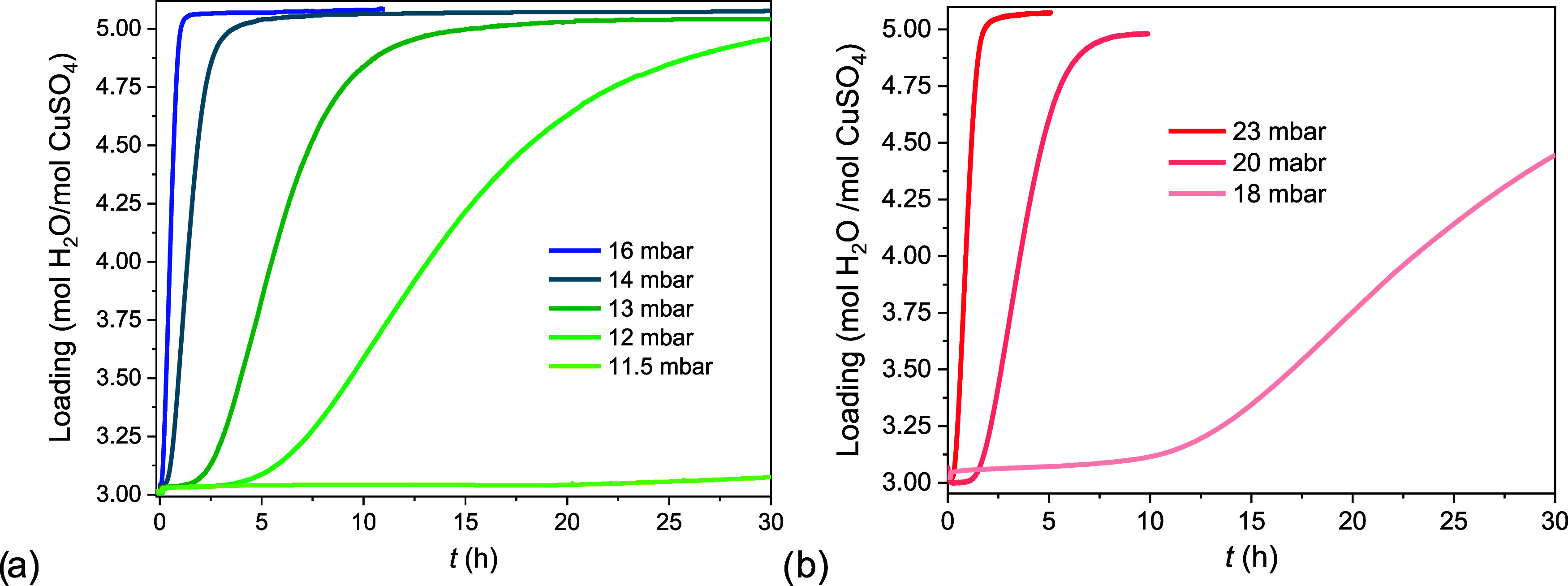
Hydration kinetic measurements of the trihydrate to pentahydrate reaction at (a) 21 °C and (b) 25 °C, at several water vapor pressures. The water uptake is expressed as loading and changes from 3 to 5 as a function of time.

In Figure 6a the hydration kinetics of C3H at 21 °C and different water vapor pressures are shown. All hydration curves were measured in the 3–5 MSZ. These were characterized by an initial period in which the loading did not change, followed by a second period in which the loading increased. These time delays, known as induction times, decreased when the water vapor pressure increased and approached zero at the 3–5 MSZ. In fact, at 16 mbar, it was only few minutes long. At 11.5 mbar, instead, it was 20 h long. Therefore, total hydration times can be extended considerably when there are time delays in the water uptake due to metastability.

We find similar characteristics when the temperature is increased to 25 °C, Figure 6b. All curves were measured within the 3–5 MSZ. At 18 and 20 mbar the induction time was in the order of hours, followed by a rapid increase in mass afterward. Closer to the MSZ boundary, 23 mbar, the induction time was of the order of minutes. At both temperatures and in the MSZ the induction time consisted of hours or tens of hours and represented a significant part of the total hydration time.

In conclusion, based on previous experimental observations,^5,10,13,48^ we could discriminate three fundamental processes in these measurements. First, the metastability of C3H, identified by the delay in water uptake. Second, the nucleation of C5H, visible in the exponential mass increase. Third, the growth of C5H, here characterized by a rather linear mass increase.

### Hydration Kinetics of CuSO_4_·H_2_O to CuSO_4_·5H_2_O

**Figure 7:**
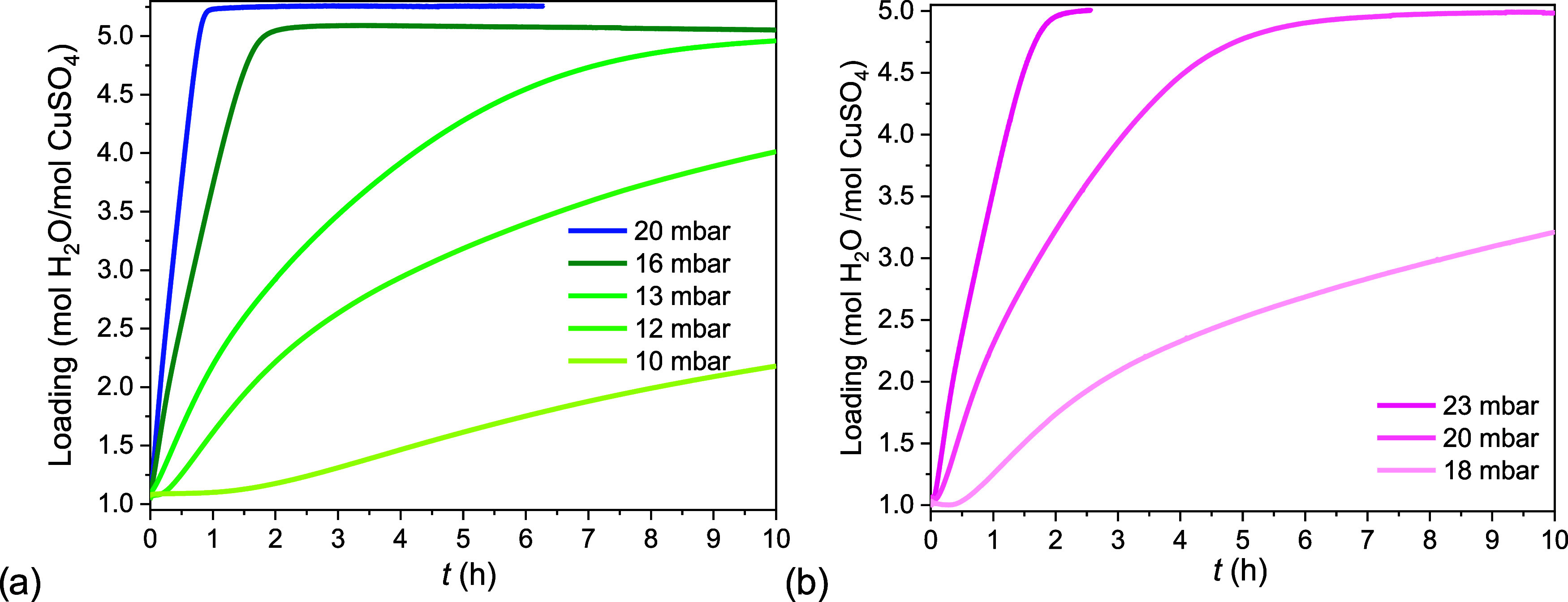
Hydration kinetic curves of the monohydrate to pentahydrate reaction at (a) 21 °C and (b) 25 °C and several water vapor pressures. The water uptake is expressed as loading and changes from 1 to 5. See Figure A5 of the Supporting Information for an enlargement of the curves around zero.

The hydration kinetics of C1H are also examined with respect to water vapor pressure and temperature (pathway 1). Since the starting phase is C1H, it is important to visualize the measurement conditions with respect to both MSZ lines, 3–5 and 1–3–5. The TGA measurement method is again isothermal–isobaric, characterized by a first mass equilibration period of 2 h at 105 °C and 5 mbar, followed by a second equilibration period of 1 h at the measurement temperature (21 or 25 °C). At *t* = 0 the water vapor pressure was quickly switched to the desired value. This time the loading varies from 1 to 5 as a function of time, as in Figure 7.

The hydration kinetic curves measured for the reaction of C1H at 21 °C are plotted in Figure 7a. Below the 1–3–5 MSZ line (10, 12, and 13 mbar), curves displayed a time delay–induction time—before the mass increased. Above the 1–3–5 MSZ line (16 and 20 mbar), curves showed an immediate response to the increase in water vapor pressure. The induction times and reaction times decreased as the water vapor pressure increased. A second induction time was never clearly observed; however, the mass uptake did not progress linearly. Due to the time scale of the figure, this nonlinearity is most evident at low water vapor pressures, below the 1–3–5 MSZ line.

The hydration kinetic curves measured at 25 °C, Figure 7b, showed similar characteristics. Below the 1–3–5 MSZ line (18 mbar), the curve had an induction time, which was not present at higher water vapor pressures (20 and 23 mbar), above that MSZ line. Again, a nonlinear mass uptake followed the induction period.

The most striking characteristic of these hydration profiles was the nonlinear mass uptake that followed the induction time. Upon closer examination of each curve (Figure 7), the uptake could be segmented into two distinct periods, with a transition occurring at a loading below 3 mol mol^–1^, where the rate of mass uptake suddenly decreased. This is also easily visualized by plots of the derivative of the loading in time (Figure A6), which are characterized by a peak, followed by a plateau. A clear induction time associated with the metastability of C3H cannot be designated. However, this transition point might be connected to the moment in which C5H is nucleated for the first time.

Investigation of the phase transformation by a bulk technique could help the association of the specific features of these curves, such as the onset point of the second period, to the determined processes. Therefore, in the next section the two hydration reactions are compared also through in situ XRD.

### Nucleation and Growth Processes during Hydration

**Figure 8:**
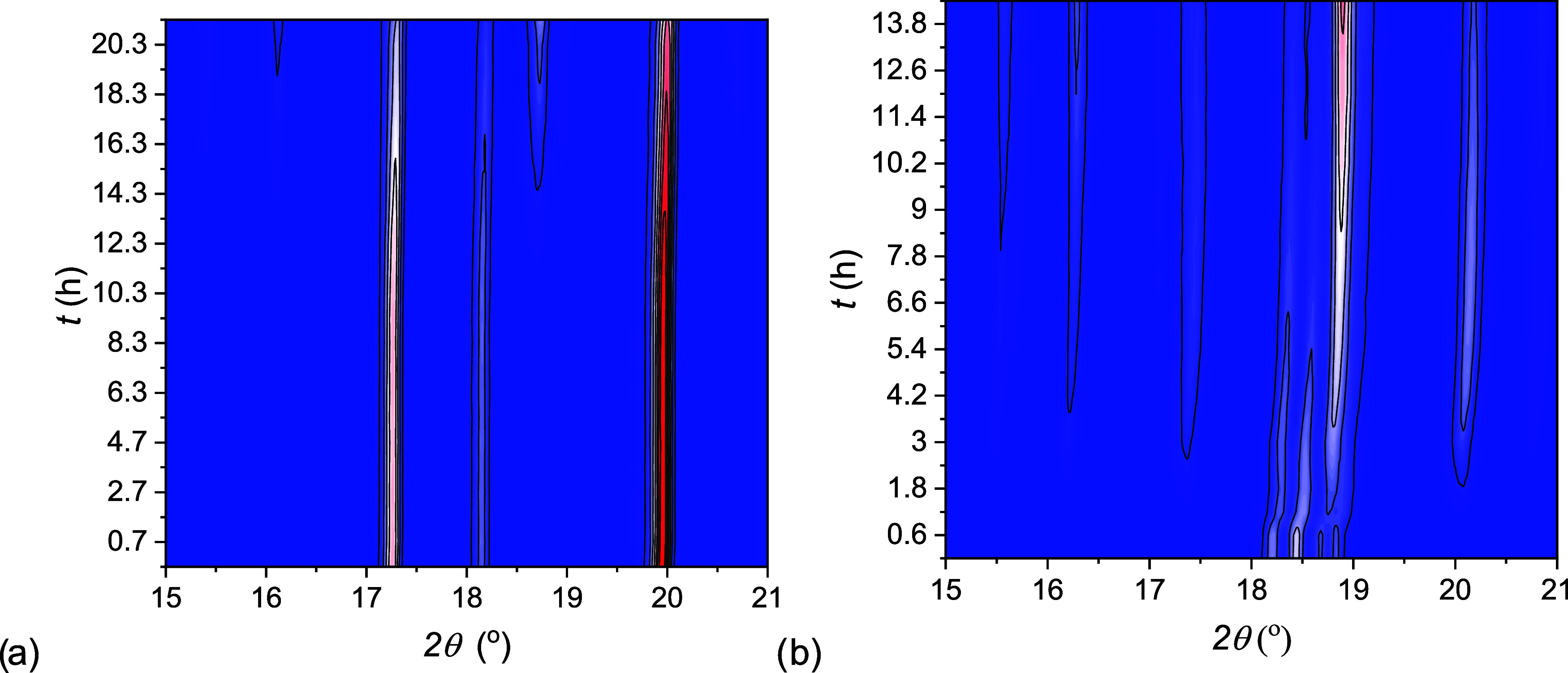
Phase composition studied by X-ray diffraction at 25.5 °C and 18 mbar as a function of time. Diffractograms collected from the in situ hydration of C3H (a) and from the in situ hydration of C1H (b). At *t* = 0, air mixed with water vapor at 18 mbar is flushed into the sample chamber. The intensity increases with the color, from blue to white to red.

The hydration mechanisms of C3H (pathway 3) and C1H (pathway 1) to C5H are investigated by in situ XRD at 25 °C and 18 mbar, therefore in coincidence with the 1–3–5 MSZ line. After an initial equilibration period, as explained in the Materials and methods, water vapor was introduced in the sample chamber and from that moment diffractograms were collected every 30–35 min. The sequence of appearance and disappearance of each phase was recorded by tracking few specific reflections, 16.3 and 18.7° (C5H), 20.3°(C3H) and 26.4° (C1H). These sequences are shown for the interval 15–21° in Figure 8 and for the interval 23–30° in Figure A7a,b of the Supporting Information.

**Figure 9:**
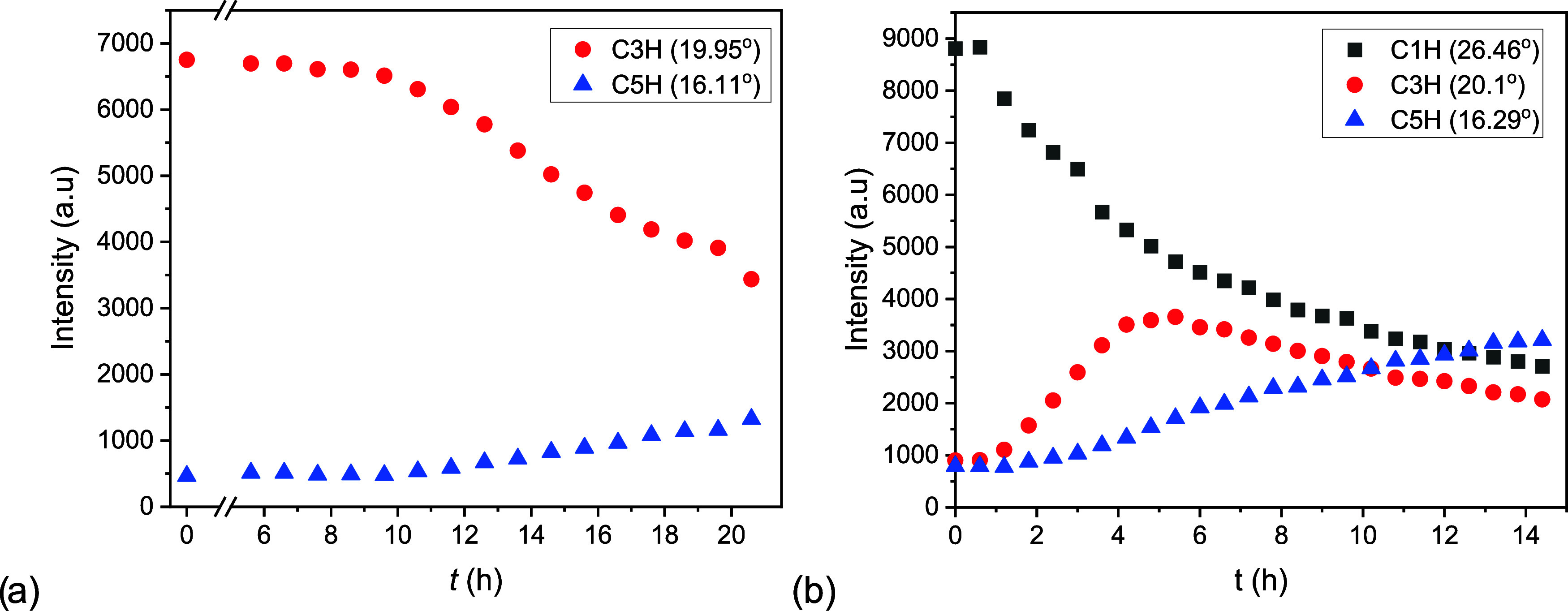
Peak intensity of selected reflections as a function of time during the hydration of C3H (a) and the hydration of C1H (b) at 25 °C and 18 mbar. Note the difference in time axis.

During the hydration of C3H (Figure 8a) reflections belonging to C5H appeared after 12 h (18.7°) and 14 h (16.3°). During C1H hydration, Figure 8b, the first reflections of C3H (20.3°) and C5H (18.7°) appeared instead after 1 h. In order to emphasize the sequence of appearance, the peak intensities of these reflections are plotted as a function of time in Figure 9.

During the hydration of C3H, the intensity of the C5H reflection increased while the intensity of the C3H reflection decreased. During C1H hydration, the intensity of C1H decreased from the beginning. After 1 h, C3H and C5H reflections were detected, and their intensity started to increase. After 4 h, the intensity of the C3H reflections reached a maximum.

The analysis of the intensity profile of C1H hydration reveals that C1H does not convert fully into C3H, before C3H transforms into C5H. We also noticed by comparing the two intensity profiles that C5H formed earlier when starting from C1H. Specifically, at 25 °C the time of appearance of C3H and C5H from C1H cannot be distinguished. By increasing the measurement temperature to 30 °C (Figure A7c), C3H appeared earlier than C5H.

XRD measurements confirm the existence of two mechanisms occurring one after another, distinguished by the production or consumption of C3H. Furthermore, they reveal a lower barrier for nucleation of C5H along pathway 1 compared to pathway 3.

### Process Limiting C1H Hydration to C5H

**Figure 10:**
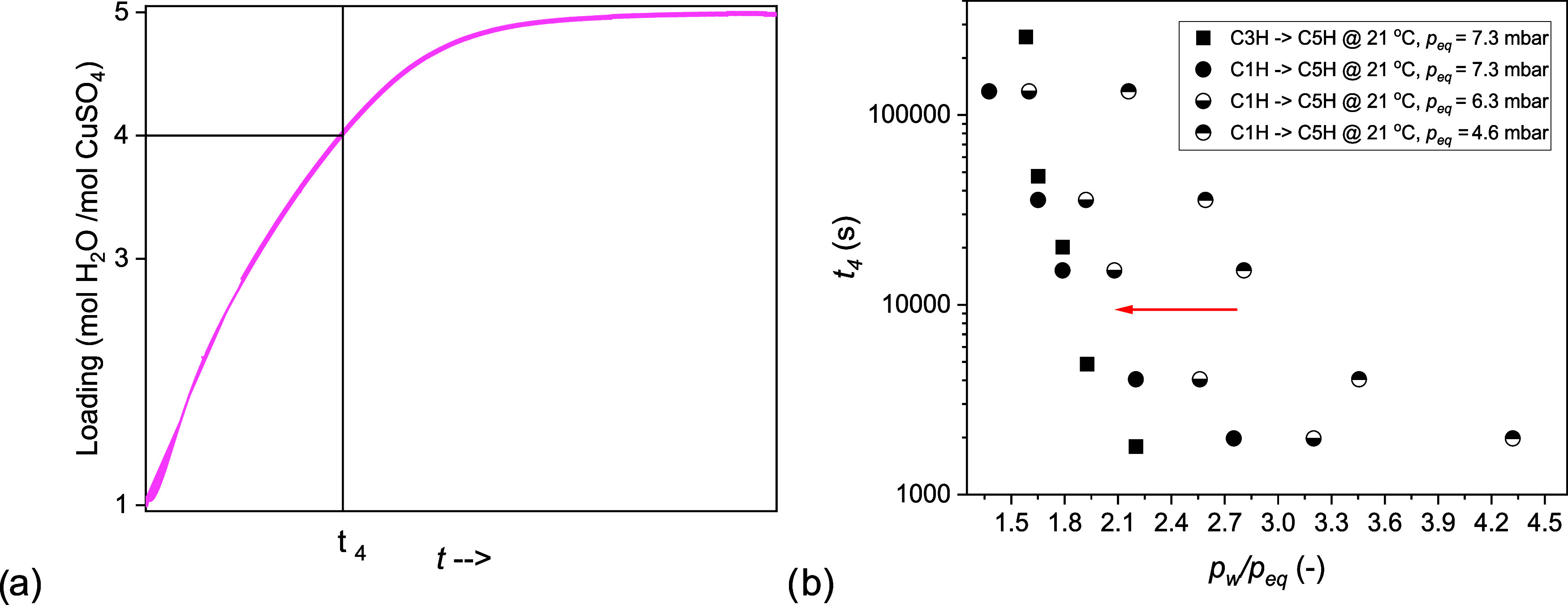
Definition of conversion time at a loading of 4 (a). Conversion time, *t*_4_ (s), as a function of the driving force, *p*_*w*_/*p*_eq_ at 21 °C (b). The conversion time is measured from the moment in which the water vapor pressure is introduced to the moment in which a loading of 4 is reached. The driving force is *p*_w_/*p*_eq_ (3–5) for the reaction from C3H to C5H (black squares). The driving force for the reaction from C1H to C5H can be either *p*_w_/*p*_eq_ (3–5) (full circles), *p*_w_/*p*_eq_ (1–3) or *p*_w_/*p*_eq_ (1–5) (half full circles). Every data point is a measurement point. The conversion times obtained from the 25 °C measurement are reported in the Supporting Information.

Analysis of the conversion times of C3H (pathway 3) and C1H (pathway 1) also gives information about the processes involved in the hydration of C1H. Specifically, we consider the conversion times obtained from the previous kinetic measurements when a loading of 4 is reached, as shown by Figure 10a, and analyze them as a function of the driving force. At a loading of 4 all processes must have started. Further we select *p*_w_/*p*_eq_ as driving force,^13^ whose character depends on the definition of *p*_eq_. In the case of C3H hydration, *p*_eq_ = *p*_3–5_(*T*), where *p*_3–5_(*T*) is the equilibrium water vapor pressure at the 3–5 equilibrium line. In the case of C1H hydration, either *p*_eq_ = *p*_1–3_(*T*), where *p*_1–3_(*T*) is the equilibrium water vapor pressure at the 1–3 equilibrium line, or *p*_eq_ = *p*_1–5_(*T*), where *p*_1–5_(*T*) is the equilibrium water vapor pressure at the 1–5 equilibrium line, or again *p*_eq_ = *p*_3–5_(*T*).

The conversion times measured at 21 °C are plotted in Figure 10b as a function of the driving force. Since three driving forces are analyzed, there are in total four curves, one referred to the hydration of C3H, three referred to the hydration of C1H. The use of a different driving force determines the curve’s position.

We found that the conversion times of C1H almost matched with the conversion times of C3H, if *p*_w_/*p*_eq_(3–5) was used to represent the data. Instead, they were shifted to the right, if either *p*_w_/*p*_eq_(1–3) or *p*_w_/*p*_eq_(1–5) was used. According to the latter cases, the conversion times for C1H hydration would be longer at any water vapor pressure. The conversion times measured at 25 °C matched as well when *p*_w_/*p*_eq_(3–5) was used to represent the data (Figure A8).

## Summary of the Mechanistic Findings

Measurements of the phase transition during hydration of C1H by Raman Spectroscopy showed the sequential transformation from C1H to C3H to C5H (Figure 6). Therefore, C1H hydrates according to pathway 1 and not pathway 2.

TGA and in situ XRD measurements revealed that pathway 1 consisted of two mechanisms: nucleation and growth of C3H and nucleation and growth of C5H.

The hydration kinetic curves of C1H (Figure 8) could be split into two intervals according to the rate, as this decreased prior to reaching a loading of 3 in every measurement. During the first interval both C3H and C5H were formed, as elucidated by XRD (Figure 10). During the second interval, C3H was predominantly consumed, while the amount of C5H still increased. For these reasons, we think that the decrease in hydration rate indicates the switch in rate limiting process, instead of the onset of a new process.

The mechanism and the kinetics are also related to the distribution of the phases in the powder particle, at the surface or in the bulk. The sharp transitions observed by Raman Spectroscopy suggest that each phase grows on top of the phase from which it nucleates, as in a shrinking core model. The inner shells are progressively less in contact with water molecules. This determined that C1H disappeared slowly from the XRD diffractograms and that the intensity of C3H reached a maximum in intensity.

**Figure 11:**
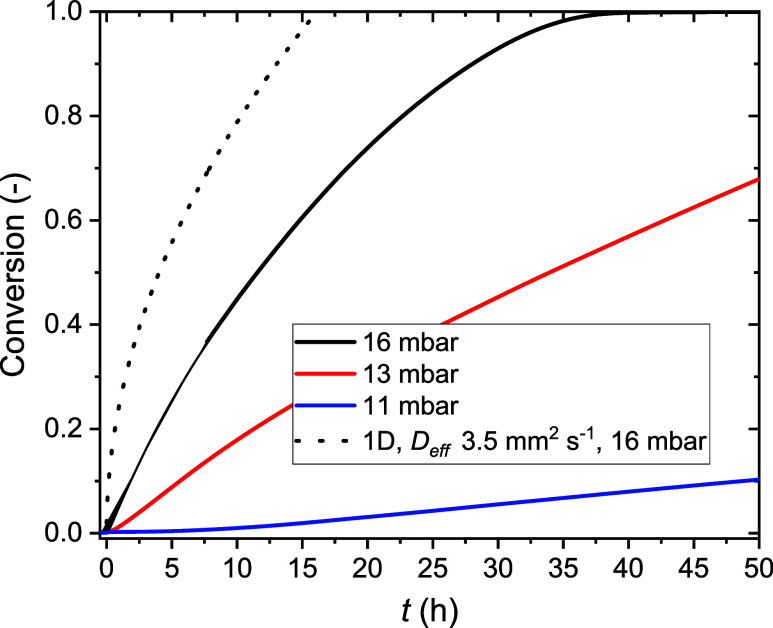
Hydration kinetic curves of CuSO_4_·H_2_O tablets pressed at 0.85 kbar (50% total porosity at the start) and measured by TGA at 21 °C and several water vapor pressures. The continuous lines represent the measurement curves. The dashed line is calculated by using the analytical solution of eq 4 from (49), for the conversion of a C1H tablet in the case of 1D diffusion limited hydration. Details of the curves at short hydration times can be found in the Supporting Information, Figure A9a.

The process limiting the hydration rate of C1H cannot be established with full certainty, but it is possible to advance some hypothesis. Based on TGA (Figure 11), the conversion times of C3H (pathway 3) and C1H (pathway 1) coincided when the same driving force was considered—*p*_w_/*p*_eq_(3–5)—which might indicate that both pathways are limited by the same rate limiting process: the mechanism of formation of C5H. Focusing on this specific mechanism, we discovered that nucleation of C5H according to pathway 3 was hindered and took several hours (XRD). While, at the same temperature and water vapor pressure conditions, nucleation of C5H according to pathway 1 took 1 h (Figure 10). The shortened induction times of C3H in pathway 1 could arise from a different morphology of the intermediate C3H compared to pure C3H and could explain the comparable conversion times.

In conclusion, three main observations—the spatial distribution of the hydrated phases, the coinciding conversion times and the shortened induction times of C3H—support the hypothesis that C1H hydration (pathway 1) might be limited by the growth of C5H.

### Consequences for Hydration of mm—Tablets

At the level of a porous salt hydrate tablet made with C1H, the description of the mechanism gains another level of complexity, because water vapor also must diffuse to the reaction site. According to Aarts et al.^50^ the hydration of tablets can be either reaction or diffusion limited. In the case of single hydration transitions, it was argued to be most probably diffusion limited.^49^ In the case of multiple hydration transitions, we do not know if this duality has to be evaluated at every hydration step or only with respect to the limiting reaction process. The hydration of C1H cylindrical tablets was therefore investigated at constant temperature and different water vapor pressures, keeping all other characteristics of the tablets (size, shape and porosity (50%)) identical. Hydration measurements are shown in Figure 11 in a plot of the conversion as a function of time at 21 °C.

According to our results, the hydration kinetics of these tablets increased with the water vapor pressure. Surprisingly, below the 1–3–5 MSZ (13 and 11 mbar), the curves displayed a time delay during which the mass did not increase, which was not present above the 1–3–5 MSZ (16 mbar). This observation strongly relates to our previous analysis of C1H hydration. Since at the powder level the induction time was linked to the metastability of C1H, also this delay in mass uptake could be related to it.

During the time delay, water vapor probably diffuses into the tablet, but the reaction does not take place because C1H is metastable. At the end of the time delay, nucleation of C3H might occur homogeneously in space, throughout the thickness of the tablet. A reaction front might not exist in this case. In the absence of C1H metastability, a reaction front could instead form. We think that depending on the time scale of diffusion with respect to the time scales of the other processes, more than one front might form.

Since we argued that C1H hydration might be controlled by a single process—growth of C5H—in Figure 11 we compare the curve measured at 16 mbar—when C1H is not metastable—to the expected conversion of a C1H tablet (dashed black line), based on the single front 1D model of Aarts.^50^ For the calculation we used the crystal density of C1H, (ρ = 3.35 g cm^–3^), the tablet thickness, (*L* = 2.2 mm) and finally the diffusion coefficient expected for a tablet of 50% porosity^49^ (*D*_eff_ = 3.5 mm^2^ s^–1^). We find that the model largely overestimates our measurement. Figure A9b in the Supporting Information shows the fit to the curve when *D*_eff_ = 1 mm^2^ s^–1^, assuming a higher tortuosity in the tablet. The fit shows agreement at conversions below 0.4. Above 0.4 the fit underestimates the measurement results.

## Conclusion

In the experiments carried out in this work, the hydration of CuSO_4_·H_2_O to CuSO_4_·5H_2_O proceeded with formation of the intermediate phase CuSO_4_·3H_2_O. Furthermore, it was revealed that the hydration kinetics of CuSO_4_·3H_2_O and CuSO_4_·H_2_O might have the same limiting reaction process: the growth of the CuSO_4_·5H_2_O phase.

Based on our work and on measurements of hydration kinetics in literature, the hydration of other salts might involve the formation of intermediate hydrate phases. In particular, the existence of nucleation and growth processes in sequence and the identification of the rate limiting process should be evaluated for other hydration reactions across multiple phases. Understanding the underlying processes directly impacts the strategies adopted to increase the power output of the reaction. For example, the metastability of C3H was substantially reduced when starting the reaction from C1H. Therefore, a proper strategy should aim at affecting growth, instead of nucleation process. A strategy could also aim at reducing the metastability of C1H, but this would have a limited benefit and could also produce unexpected effects, as the MSZ line could be moved outside of the thermodynamic stability region of C5H.

Many questions regarding the details of the mechanism remain unanswered. It would be relevant to investigate these phases grow on top of each other at the level of a powder particle. The morphology of the intermediate C3H may have an impact on the hydration kinetics of C1H which could be investigated by varying the dehydration conditions (temperature, water vapor pressure, atmospheric conditions) at which C1H and C3H are prepared. Factors such as porosity, surface area, particles size distribution may affect the hydration kinetics. In Figure A10 of the Supporting Information we show for example that the second period of C1H hydration is strongly affected by powder size distribution.

Knowledge of sequential processes during hydration is also useful to the characterization of the hydration kinetics of mm-size materials, such as tablets and composites. According to recent publications,^50^ the hydration kinetics of these systems depends on the speed of powder hydration with respect to water diffusion. We have shown that for C1H tablets the current 1D front diffusion model is not sufficient to describe the reaction kinetics and we have argued that the complexity of C1H hydration might result into formation of more than one front.

So far, hydrating across multiple phases has been deemed desirable because of the higher energy density. This benefit is already counterbalanced by the occurrence of even larger volume variations—caused by the absorption of a larger number of water moles. Now we also need to question if the power output benefits from hydrating across multiple phase transitions. On one hand, at low hydration levels the power output is the highest. On the other hand, growth of the higher hydrate species determines the total hydration rate.

## Supporting Information Available

SEM images, figures showing the complete measurement in support to data points shown in the main text (EIS, TGA) and additional measurements in support to the discussion in the main text (XRD) (PDF)
