# Supplementary material for: Mechanism and Kinetics of Hydration of CuSO4·H2O in the Presence of an Intermediate Step
Source: Cryst Growth Des. 2024 Dec 9;24(24):10082–93. doi: 10.1021/acs.cgd.4c00589 (PMC11660158; doi:10.1021/acs.cgd.4c00589)
Supplement: Supplementary file 1 — cg4c00589_si_001.pdf [file cg4c00589_si_001.pdf]

Mechanism and kinetics of hydration of $CuSO_4 \cdot H_2O$ in presence of an intermediate step

Martina Cotti [a,b], Amelie Stahlbuhk [c], Hartmut R. Fischer [d], Michael Steiger [c], Olaf C.G. Adan [b,d] Henk P. Huinink [a,b*]

[a] Eindhoven Institute of Renewable Energy Systems, Eindhoven University of Technology, PO Box 513, Eindhoven 5600 MB, the Netherlands
[b] Transport in Permeable Media group, Department of Applied Physics, Eindhoven University of Technology, PO Box 513, Eindhoven 5600 MB, the Netherlands
[c] Department of Chemistry, University of Hamburg, Martin-Luther-King-Platz 6, 20146 Hamburg, Germany
[d] TNO Materials Solution, High Tech Campus 25, 5656 AE, Eindhoven, Netherlands

Nomenclature

ΔS, entropy of dehydration (J/mol/K)
ΔH, enthalpy of dehydration (kJ/mol)

RS, Raman spectroscopy

C1H, $CuSO_4 \cdot H_2O$
C3H, $CuSO_4 \cdot 3H_2O$
C5H, $CuSO_4 \cdot 5H_2O$

EIS, electrochemical impedance spectroscopy

MSZ, metastable zone

p_{eq} equilibrium water vapor pressure (mbar)
p_w, water vapor pressure (mbar)
R, ideal gas constant (J/mol/K)
RH, relative humidity (%)
T, temperature (K)

TGA, thermal gravimetric analysis

XRD, x-ray diffraction

Supplementary Information

A1. Secondary electron images of the starting substances

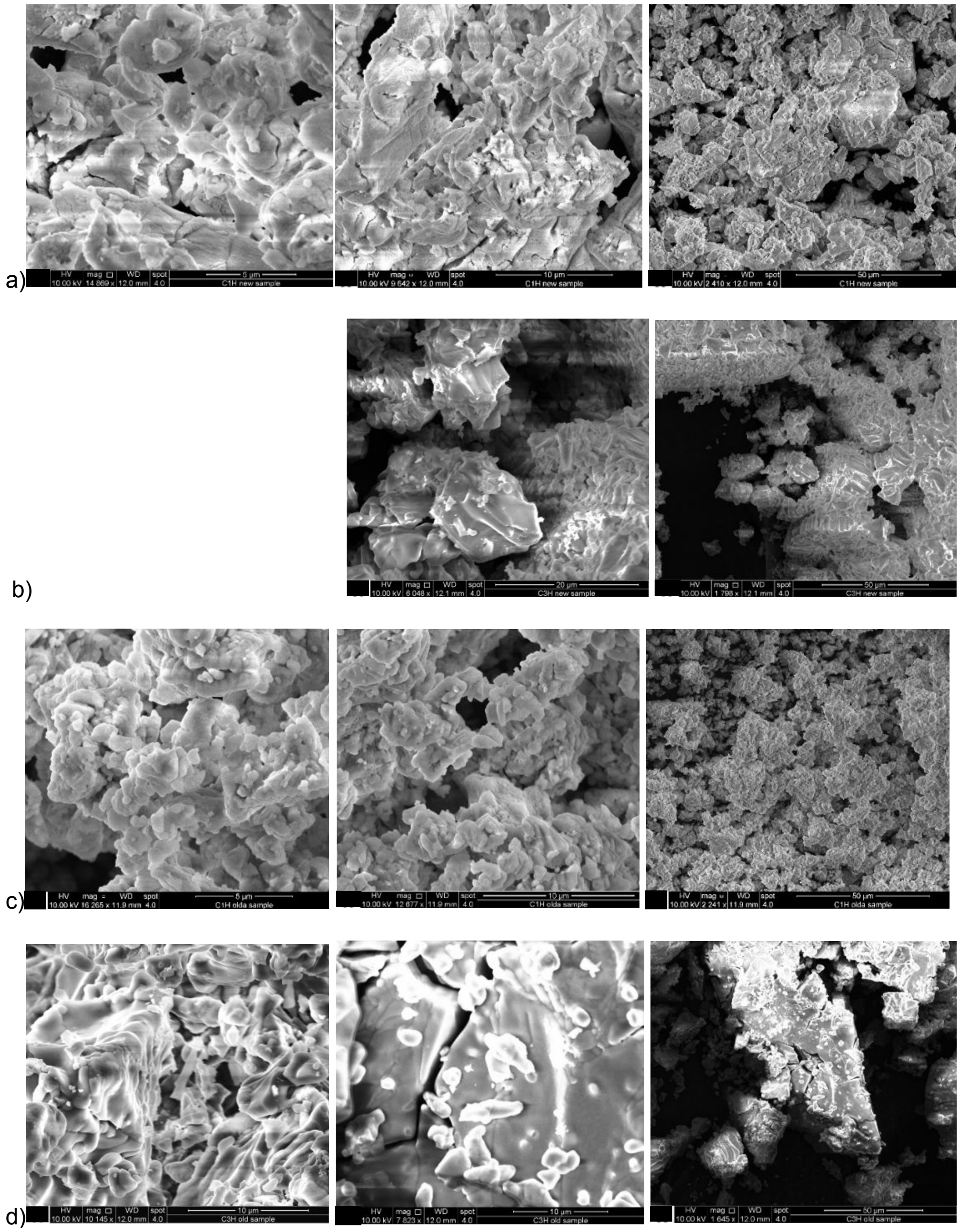

Figure A 1: Secondary scanning electron images of $CuSO_4{\cdot}H_2O$ (a-c) and $CuSO_4{\cdot}3H_2O$ (b-d) obtained from d < 50 μm (a-b) and from 50<d<150 μm (c-d) $CuSO_4{\cdot}5H_2O$ sieved powder by dehydration at 105°C and 40°C, respectively.

A2. Measurements of hydration and dehydration onset temperatures

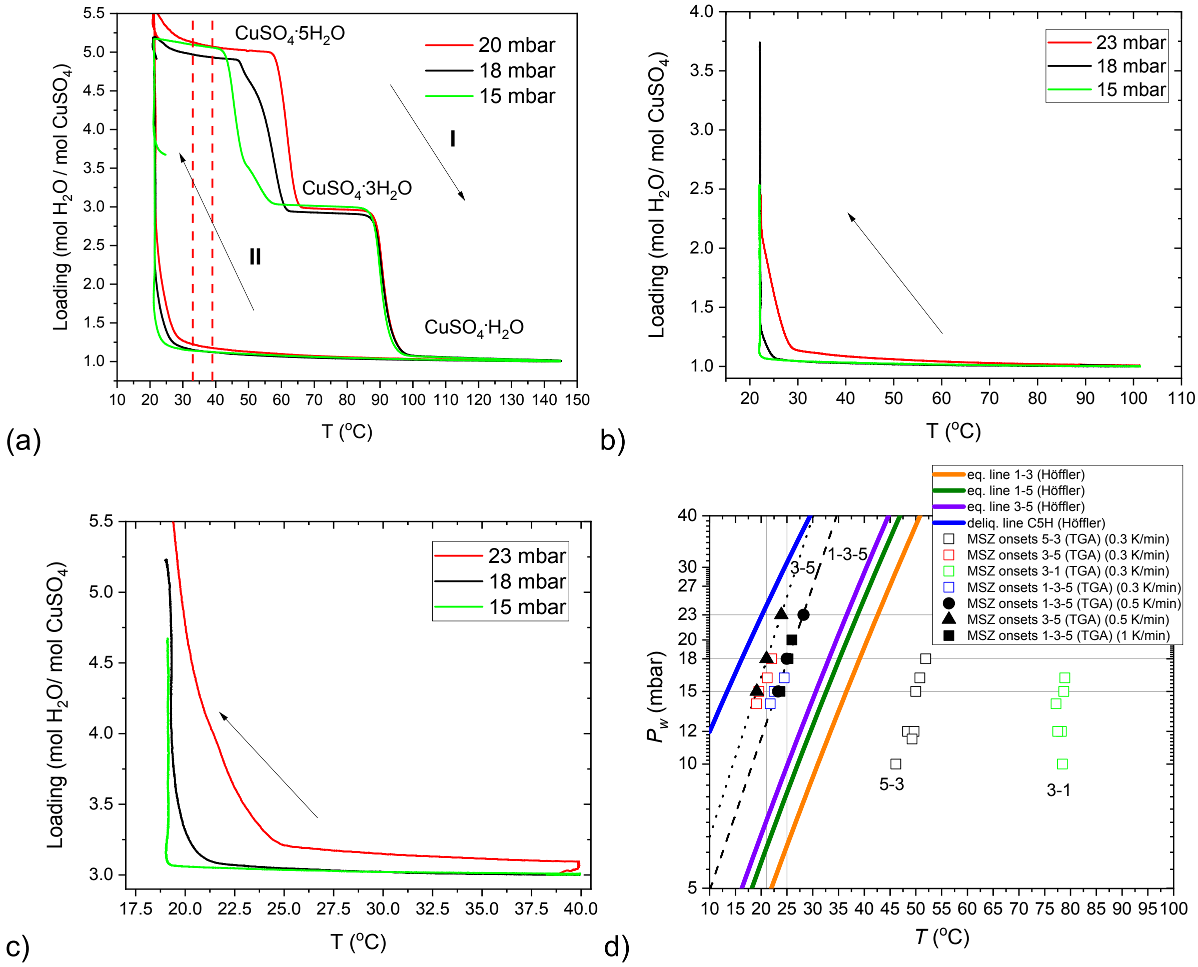

Figure A 2: Isobaric measurements of the metastable zone onset temperatures by TGA, starting from C5H (1 K/min) (a), C1H (0.5 K/min) (b) and C3H (0.5 K/min) (c). (d) Phase diagram of $CuSO_4$ showing the metastable zone measurement points for the hydration from C1H to C5H (blue open squares, 0.3 K/min), from C3H to C5H (red open squares, 0.3 K/min), as well as the metastable zone measurement points for the dehydration from C5H to C3H (black open squares, 0.3 K/min) and from C3H to C1H (green open squares, 0.3 K/min).

A3. Impedance spectroscopy measurements of the metastable zones

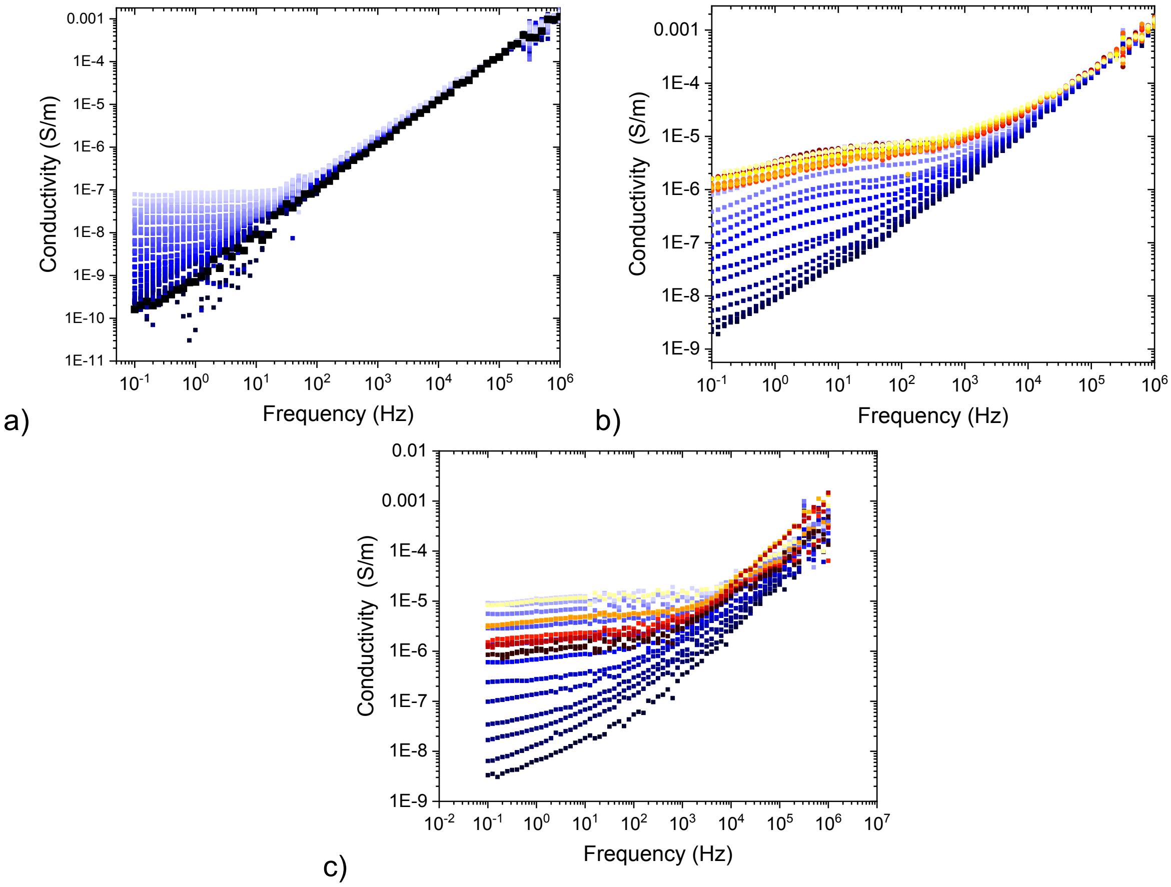

Figure A 3: Measurements of the conductivity (S/m) as a function of frequency (Hz) at different water vapor pressures and 25°C for C5H (a), C3H (b) and C1H (c). Initially the admittivity increases with the water vapor pressure – dark blue to light blue spectra. At a certain value of water vapor pressure, the admittivity begins to drop – yellow to red spectra.

A4. Nucleation on the particles' surface

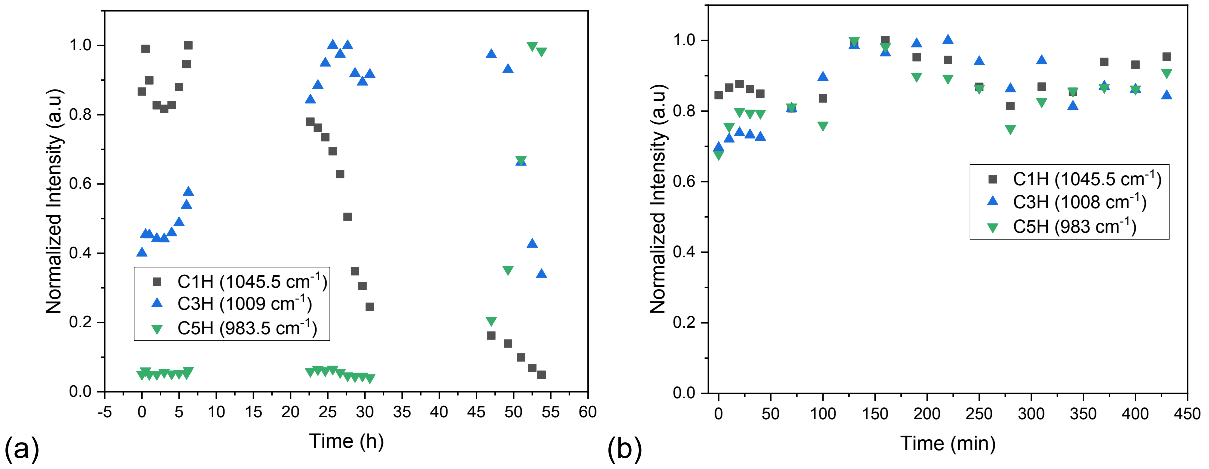

Figure A 4: Normalized intensity of the vibrations measured by confocal Raman microscopy as a function of time, at 60% RH (a) and 40% RH (b) and 21 °C.

A5. The hydration kinetics

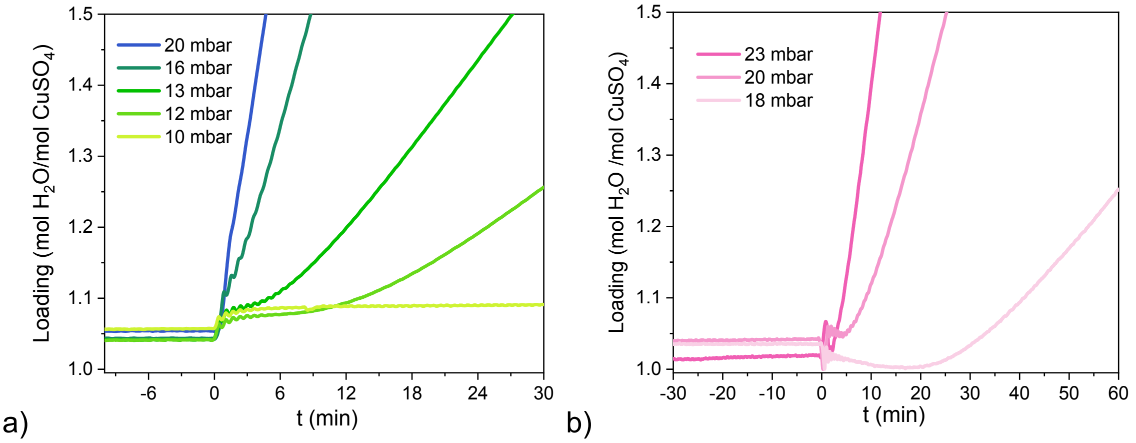

Figure A 5: Enlargement of the hydration kinetic curves of the monohydrate to pentahydrate reaction at (a) 21 °C and (b) 25 °C and several water vapor pressures. The water uptake is expressed as loading and changes from 1 to 5.

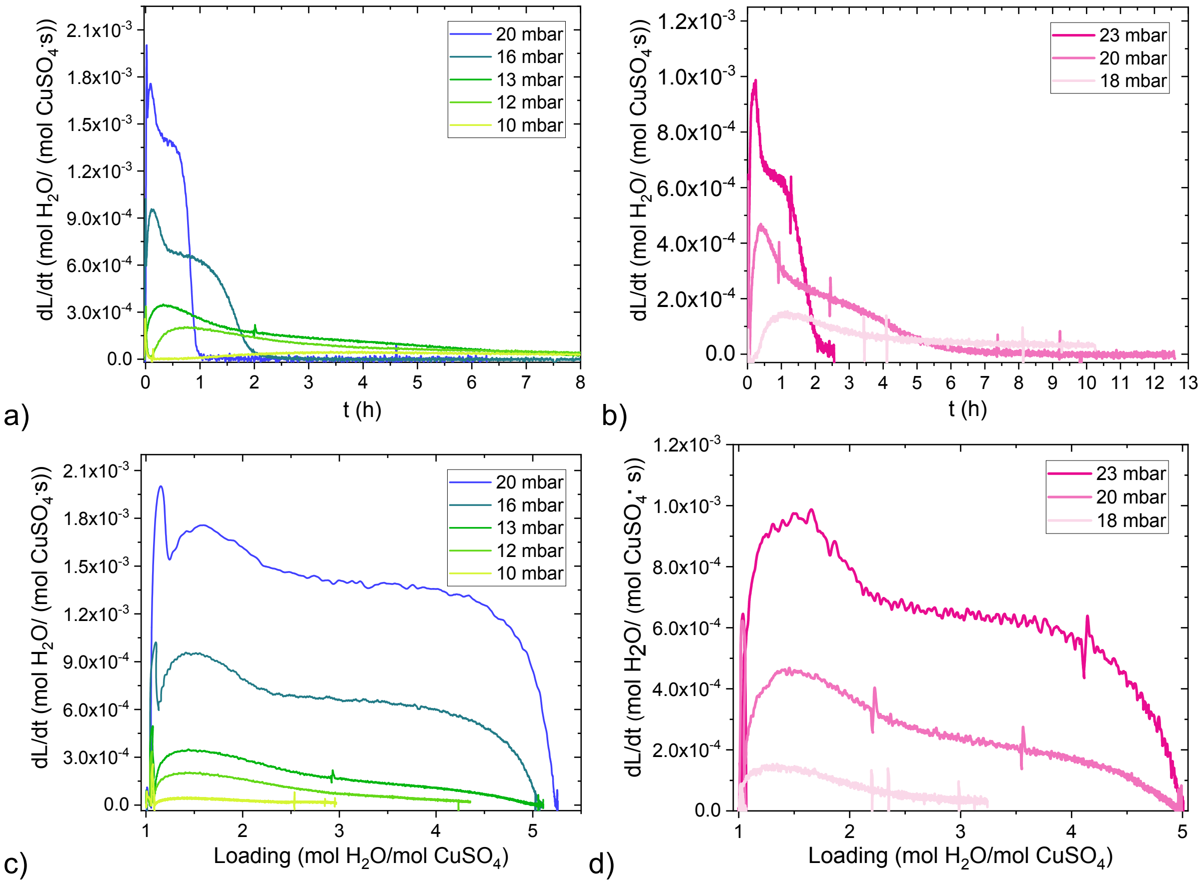

Figure A 6: First time derivative of the loading as a function of time (a-b) and as a function of loading (c-d) calculated from the hydration kinetic measurements at 21°C (a-c) and 25 °C (b-d). Both representations emphasize an initial increase in rate, up to a loading of 2, followed by a long plateau, during which the rate is constant before rapidly falling to zero at the end of the reaction. The spike at the beginning of every measurement is an artifact caused by buoyancy, at the moment in which the water vapor is introduced in the chamber.

A6. Phase transformation during hydration (XRD)

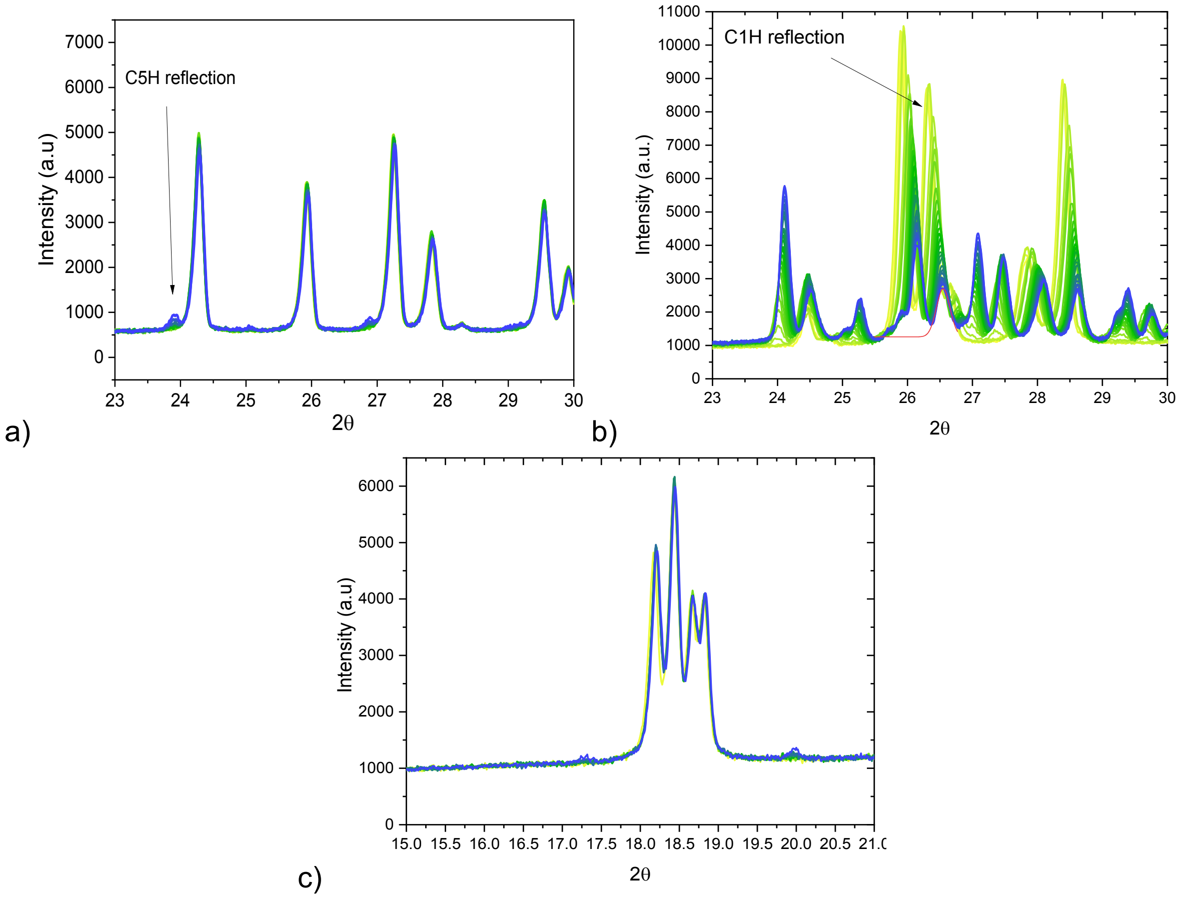

Figure A 7: Phase composition studied by X-ray diffraction during the in-situ hydration of C3H at 25.5 °C and 18 mbar (a) and C1H at 18 mbar and 25.5 °C (b) and 30°C (c). At t=0, air mixed with water vapor at 18 mbar is flushed into the sample chamber. The color of the diffractograms gets darker as t increases (yellow-green-blue)

A7. Conversion time and driving force

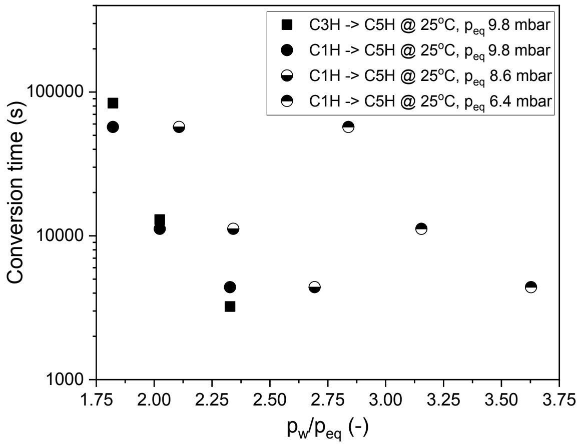

Figure A 8: Conversion time (s) as a function of the driving force, p_w/p_{eq}, at 25°C.

A8. Hydration kinetics of tablets

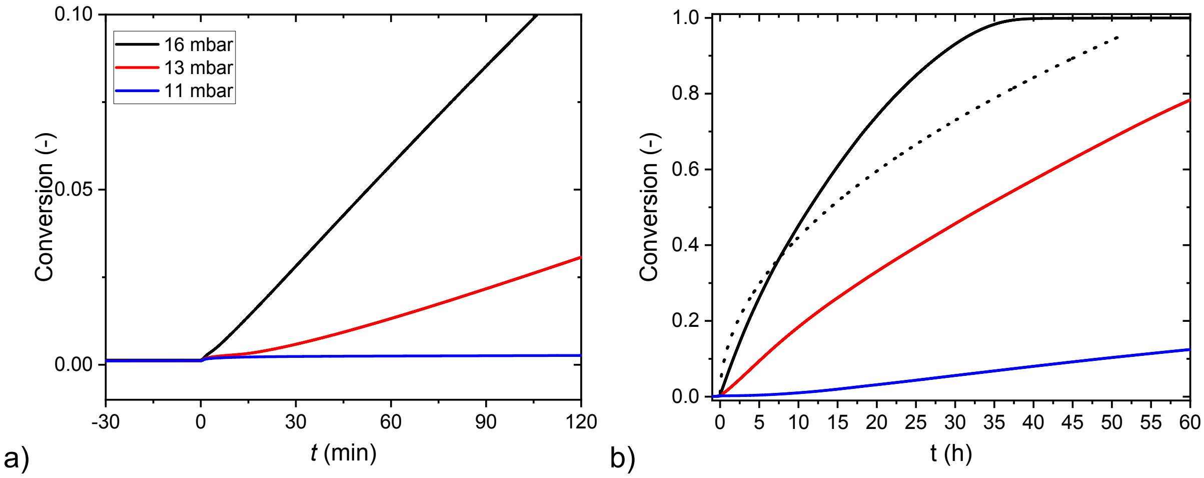

Figure A 9: (a) Detail of the hydration kinetic curves of $CuSO_4 \cdot H_2O$ tablets pressed at 0.85 kbar (50% total porosity at the start) and measured by TGA at 21 °C and several water vapor pressures.(b) The dashed line is calculated by using the analytical solution of equation 4 from (Cotti et al.) for the conversion of a C1H tablet in the case of 1D diffusion limited hydration, using a D_{eff} = 1 mm^2s^{-1}.

A9. Hydration kinetics as a function of particle size

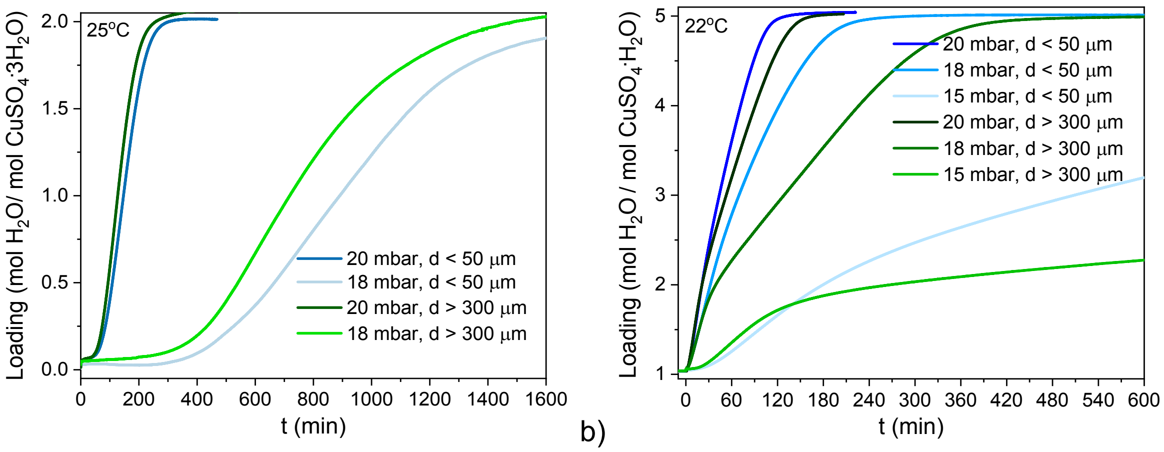

Figure A 10: Hydration kinetic curves of copper sulfate trihydrate (a) and monohydrate (b) with varying powder particle size, either > 300 μm (green curves) or < 50 μm (blue curves). The measurements are conducted at constant temperature (25 and 22°C) and several water vapor pressures.
